# Antitumor and Immunomodulatory Effects of Polysaccharides from Broken-Spore of *Ganoderma lucidum*

**DOI:** 10.3389/fphar.2012.00135

**Published:** 2012-07-13

**Authors:** Peng-Yun Wang, Xiao-Ling Zhu, Zhi-Bin Lin

**Affiliations:** ^1^Department of Pharmacology, School of Basic Medical Sciences, Peking University Health Science CenterBeijing, China

**Keywords:** *Ganoderma lucidum*, spores, polysaccharide, antitumor activity, cytokines, lymphocytes, NK cells, macrophages

## Abstract

The antitumor and immunomodulatory activity of broken-spore of *Ganoderma* *lucidum* polysaccharides (*Gl*-BSP) were investigated *in vivo* and *in vitro*. It was showed that *Gl*-BSP (50, 100, and 200 mg kg^−1^) exhibited antitumor effect against Sarcoma 180 (S180) in BALB/c mice. The *Gl*-BSP was not cytotoxicity in S180 cells and PG cells (human lung carcinoma cell) *in vitro*. However, serum from *Gl*-BSP-treated S180-bearing mice significantly inhibited S180 and PG cells proliferation *in vitro*. Moreover, *Gl*-BSP promoted the splenic lymphocyte proliferation induced by Con A or LPS, enhanced nature killer cell (NK cell) cytotoxic activity, augmented the percentage of neutral red phagocytosis by macrophages, and increased the percentage of the CD4^+^ or CD8^+^ subset in S180-bearing mice. The serum level of IFN-γ, TNF-α, and nitric oxide was increased by *Gl*-BSP. *Gl*-BSP also showed immunomodulatory activities in tumor-bearing mice. Furthermore, neutralization with anti-TNF-α and/or anti-IFN-γ significantly diminished growth inhibition induced by *Gl*-BSP-treated serum of S180-bearing mice in S180 or PG cells. These observations suggest that the antitumor activity of *Gl*-BSP may be mainly related to the activation of the immune response of the host organism by the stimulation of NK cells, T cells, and macrophages.

## Introduction

*Ganoderma lucidum* (Leyss. et Fr.) Karst. (*G. lucidum*) is widely used in China and other oriental countries (Lin, 2001). *G. lucidum* (Lingzhi or Reishi) has been reported to be effective in modulating immune functions, inhibiting tumor growth (Sliva et al., 2002; Susan et al., 2002; Lin and Zhang, 2004), preventing oxidative damage (Kim et al., 1999; You and Lin, 2002), protecting liver, and reducing serum glucose levels, along with no toxicity (Zhang et al., 2002; Zhang and Lin 2003).

Polysaccharides are one of active component of *G. lucidum*. Antitumor effects of polysaccharides isolated from *G. lucidum* were originally observed in sarcoma 180 bearing mice (Miyazaki and Nishijima, 1981; Sone et al., 1985). Recently, the antitumor effects of *G. lucidum* polysaccharides have been extensively investigated and were mainly through immune-related mechanisms (Xia et al., 1989; Sakagami et al., 1991; Wang et al., 1997, 2002; Hu and Lin, 1999; Yan et al., 1999; Zhang and Lin, 1999; Hsu et al., 2002, 2003; Wasser, 2002; Lee et al., 2003).

Recently, the spores of *G. lucidum* have attracted much attention for their versatile biological activities (Yang et al., 1997; Liu, 1999; Zhu et al., 2000; Liu et al., 2002; Lu et al. 2004). Bao et al. (2000, 2001a,b) has isolated several polysaccharides from broken or non-broken spores of *G. lucidum*, and proved these polysaccharides have immunomodulatory activities. The release ability of polysaccharides of broken spores has much greater than that of non-broken spores (Bao and Fang, 2001). In this study, we examined the antitumor activity of broken spore polysaccharides (*Gl*-BSP) extracted from *G*. *lucidum*, and investigated the potential antitumor mechanism of *Gl*-BSP.

## Materials and Methods

### Animals

Inbred male 6–8 weeks old (body weight 18–22 g) BALB/c (H-2 d) mice (Grade II, Certificate No scxk11-00-0004) were purchased from the Department of Experimental Animals, Health Science Center, Peking University, Beijing, China. All procedures were in accordance to the Institute Ethical Committee for Experimental Use of Animals.

### Cell lines

*Murine* Sarcoma 180 cell lines (S180) were obtained from Beijing Tumor Institute. Human lung carcinoma cell lines (PG) and YAC-1 cell lines were provided by Department of Pathology and Immunology, Peking University Health Center. The cells were maintained in RMPI-1640 supplemented with 10% fetal calf serum (FCS) in a humidified environment at 37°C and 5% CO_2_, and cultures were passaged every 2 or 3 days.

### Drugs

*Gl*-BSP was kindly provided by Fuzhou Institute of Green Valley Bio-Pharm Technology. It was a sandy beige water soluble powder and was isolated from boiling water extract of the broken-spore of *G. lucidum* (Leyss. et Fr.) Karst. (*Gl*), followed by ethanol precipitation, dialysis, and protein depletion using Sevag method. The purity of the *Gl*-BSP was 97%, which was analyzed by high-performance liquid chromatography (HPLC). The component sugars and molecular-weight distributions of the glycopeptides were determined by gel permeation chromatography (GPC) and HPLC. The structures of the glycopeptides were detected by IR, ^1^H NMR, and ^13^C NMR. *Gl*-BSP had a molecular weight of 512,500 with a ratio of polysaccharides to peptides of 94.8:5.2%. The polysaccharides consisted of d-rhamnose, d-xylose, d-fructose, d-galactose, and d-glucose with a molar ratio of 0.549:3.614:3.167:0.556:6.89 linked together by β-glycosidic linkages. The peptides contained the following 16 amino acids: Asp, Thr, Ser, Glu, Gly, Ala, Cys, Val, Met, Ile, Leu, Phe, Lys, His, Arg, and Pro. *Gl*-BSP was dissolved in physiological saline for *in vivo* experiments or in serum-free RPMI-1640 (Gibco Laboratories, Grand Island, NY, USA) for *in vitro* experiments, filtered through a 0.22 μm filter and stored at 4°C for future use. Endotoxins concentration in *Gl*-BSP samples were assayed under endotoxin-free experimental conditions by using a limulus amebocytes lysate chromogenic assay kit (Beijing BXGK Technology Development Co., Ltd., Beijing, China) according to the manufacture’s instruction. The quantity of endotoxin in *Gl*-BSP was less than 0.01 EU mg^−1^, indicating that endotoxin contamination in *Gl*-BSP was negligible.

### Antitumor experiment in tumor-bearing mice

Murine Sarcoma 180 cell lines were injected into peritoneal cavity of mouse, proliferated there to produce ascites, and were maintained by weekly transplantation of the tumor cells from ascites into another peritoneal cavity of mouse. S180 were taken out from the ascites, adjusted concentration as 1 × 10^7^ cells ml^−1^ with physiological saline, and then injected in 0.2 ml (2 × 10^6^ cells) into the axillary fossa of a mice right foreleg to prepare tumor-bearing mice. The 50 mice were divided into five groups randomly (10 mice in each group) 24 h after the tumor inoculation and treated for 14 consecutive days with (1) low-dose *Gl*-BSP (50 mg kg^−1^) once daily by gavage, (2) intermediate-dose *Gl*-BSP (100 mg kg^−1^) once daily by gavage, (3) high-dose *Gl*-BSP (200 mg kg^−1^) once daily by gavage, (4) cyclophosphamide (CY, an antitumor agent, 30 mg kg^−1^) once every other day by intraperitoneal injection as positive controls, and (5) sterile physiological saline (10 ml kg^−1^) once daily by gavage as negative controls (model group), and the sixth group of normal mice administrated sterile physiological saline intragastrically once daily (10 ml kg^−1^) served as normal control.

After completion of treatment on the 14th day, the mice were killed and the tumors were removed and weighted. At the same time, blood samples were collected from the orbital vein and the serum was sterilized by filtration and preserved at −70°C.

### PG cells and S180 cells proliferation assay

PG or S180 cells were maintained in RMPI-1640 supplemented with 10% FCS. A cell suspension (2 × 10^7^ cell per liter) was planted onto 96-well plates (0.1 ml per well), and incubated at 37°C, 5% CO_2_ for 24 h. The media was replaced with 0.1 ml of RPMI-1640 supplemented with 5% FCS and *Gl*-BSP 0.1, 1, 10, 100, or 400 mg l^−1^. In another series, RPMI-1640 was supplemented with 5% *Gl*-BSP treated serum 50, 100, or 200 mg kg^−1^. After 48 h, cells proliferation was estimated based on the cellular reduction of tetrazolium salt MTT by a microplate reader (BIO-RAD, Model 550), using a test wavelength of 540 nm.

### Preparation of spleen lymphocytes

Mice in all treated groups were killed and the spleens were aseptically removed, chopped with two slides and filtered over a fine nylon mesh to obtain single-cell suspensions. The cells were washed and lymphocytes were separated from red blood cells by Tris-HCl-buffered NH_4_Cl solution [mix 9 volumes of 0.83% NH_4_Cl with 1 volume of Tris-HCl (2.06%, pH 7.65), adjust pH 7.2]. Cells were finally suspended in 10% FCS RPMI-1640 supplemented with benzylpenicillin 100 kU l^−1^, streptomycin 100 mg l^−1^ for further experiments.

### Determination of proliferation of lymphocytes

Lymphocytes were planted into 96-well plates (2 × 10^6^ cells per well) with or without ConA 1 mg l^−1^ or LPS 5 mg l^−1^, and incubated at 37°C, 5% CO_2_ for 72 h. Cell proliferation was estimated based on the method of MTT.

### Splenic NK cytotoxic activity assay

The NK cell activity of spleen cells was determined by a 4 h ^51^Cr-release assay. Viable trypan blue-excluded lymphocytes were counted. Cell survival rate was greater than 95%. Target cells of YAC-1 were incubated for 1 h at 37°C with Na^51^CrO_4_ and were coated at a concentration of 1 × 10^4^ cells per well in 96-well U-bottomed culture plates after washing. Effectors and targets were mixed at a ratio of 40:1 and incubated for 4 h at 37°C. Spontaneous release was determined from wells that contained labeled target cells alone, and maximum ^51^Cr-release was determined by addition of 1% Triton X-100 (Sigma). The radioactivity of the supernatants was counted in γ-counter (Beckman LS 6500). Specific cytotoxicity was calculated as: percentage specific release = 100 × (cpm experimental release − cpm spontaneous release)/(cpm maximum release − cpm spontaneous release).

### Neutral red phagocytosis assay of peritoneal macrophages

Macrophages were obtained from mice peritoneal exudates cells (PECs). PECs were washed twice and resuspended in a RPMI-1640 medium containing 10% FCS. Peritoneal macrophages were further isolated from the PECs by incubating the PECs (2 × 10^5^ cells per well) in a 96-well plate at 37°C, 5% CO_2_ for 4 h in a humidified atmosphere to allow peritoneal macrophages to adhere. The supernatants were discarded, and 0.075% Neutral red (NR) were added and incubated for another 1 h. Cells were then washed with PBS for three times to remove excess dye and incubated with cell lysis buffer (1 M acetic acid: ethanol = 1:1) overnight. The absorbance (OD) was measured at 540 nm in a microplate reader (BIO-RAD, Model 550) and translated into phagocytosis ratio for comparison: phagocytosis ratio = test_OD_/normal control_OD_ × 100%.

### Measurement of splenic T lymphocyte subpopulations

The cell concentration of lymphocytes was adjusted to 1 × 0^7^ ml^−1^. A volume of 20 μl of FITC-labeled anti-mouse CD4 and 20 μl of PE-labeled anti-mouse CD8 was added to 100 μl of the cell suspension. After incubation for 30 min at 4°C, the lymphocytes were rinsed three times with 1 ml of PBS, pH 7.4, containing 0.5% BSA and 0.1% NaN_3_. CD4 and CD8 T-cell subpopulations were analyzed by flow cytometry using a FACS Calibur (Becton Dickinson).

### Assay for cytokines

Serum collected from tested mice were assayed for the level of IL-2, TNF-α, and IFN-γ using commercially available kits from R&D systems, with a solid-phase enzyme-linked immunoabsorbent assay (ELISA) as described by the manufacturers.

### Production of nitrite ion

The amount of ${\text{NO}}_{\text{2}}^{\text{−}}$ present in fresh serum collected from tested mice were assayed by Griess reagent, using NaNO_2_ as a standard. Briefly, 50 μl of serum was reacted with 50 μl of Griess reagent (1% sulfanilamide in 5% H_3_PO_4_ −0.1% naphthalene ethylendiamine dihydrochloride) for 10 min. The absorbance was then read at 540 nm using a microplate reader.

### Statistical analysis

Statistical significance was determined by one-way ANOVA employing the computer SPSS statistic package. *P* < 0.05 was considered significant.

## Results

### Antitumor effect of *Gl*-BSP on sarcoma 180 in mice

In *in vivo* experiment, mice were implanted with Sarcoma 180 and different dosages of *Gl*-BPS or physiological saline (model group) were administrated intragastrically for 14 days. Compared with the model group, *Gl*-BSP 100 and 200 mg kg^−1^ significantly inhibited growth of Sarcoma 180 by 49.1 and 59.9%, respectively, with no effect on body weight (Table 1).

**Table 1:** **Antitumor effect of *Gl*-BSP on Sarcoma 180 in BALB/c mice (*n* = 10, $\bar{x}\pm \text{s}$)**.

Group	Dose (mg kg^−1^ × days)	Body weight (g)		Tumor weight (g)	Inhibitory ratio (%)
		Origin	After	
Model	–	20.4 ± 0.5	20.4 ± 1.1	1.21 ± 0.27	0
*Gl*-BSP	50 mg kg^−1^ × 14	20.9 ± 0.9	20.4 ± 0.9	0.84 ± 0.42	30.7
	100 mg kg^−1^ × 14	20.5 ± 0.8	20.5 ± 1.4	0.61 ± 0.47*	49.1
	200 mg kg^−1^ × 14	20.9 ± 0.8	21.6 ± 0.8**	0.48 ± 0.39**	59.9
CY	30 mg kg^−1^ × 7	20.4 ± 0.5	18.2 ± 1.5	0.23 ± 0.11***	81.0

Mice were implanted with Sarcoma 180 and administrated with *Gl*-BPS (50, 100, or 200 mg kg^−1^) or physiological saline intragastrically for 14 days. At the end of experiment, the tumor were removed and weighted. *P < 0.05, **P < 0.01, ***P < 0.001 vs. model group.

### Effect of *Gl*-BSP on proliferation of sarcoma 180 and PG cells *in vitro*

**Figure 1:**
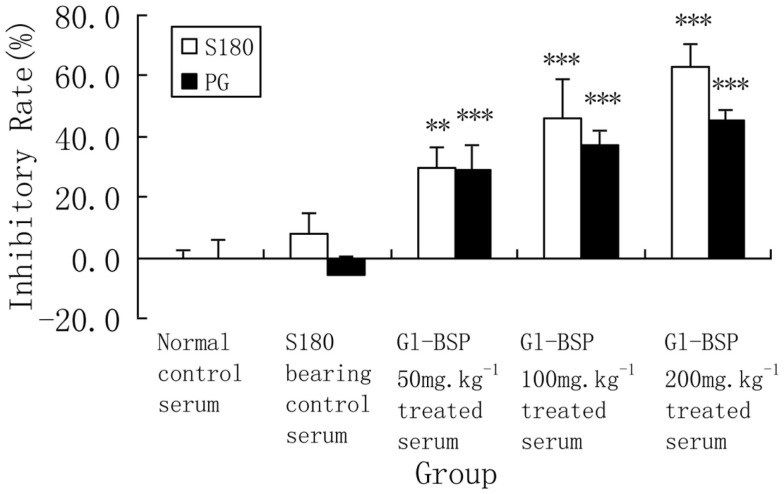
**Effect of *Gl*-BSP-treated serum on proliferation of S180 and PG cells *in vitro* (*n* = 9, $\bar{x}\pm \text{s}$)**. Mice were implanted with Sarcoma 180 and *Gl*-BPS (50, 100, or 200 mg kg^−1^) or physiological saline were administrated intragastrically for 14 days. At the end of experiment, blood samples were collected from the orbital vein and the serum was sterilized by filtration. A cell suspension (2 × 10^7^ cell per liter) was made with 5% FCS RPMI-1640, planted onto 96-well culture plates (0.1 ml per well), and incubated at 37°C, 5% CO_2_ for 24 h. The media was replaced with 0.1 ml of RPMI-1640 supplemented with 5% *Gl*-BSP treated serum 50, 100, or 200 mg kg^−1^. After 48 h, cells proliferation was estimated by MTT. Data are the mean ± SD of three separate experiments. **P < 0.01, ***P < 0.001 vs. S180-bearing control serum (model group).

*Gl*-BSP did not inhibit Sarcoma 180 and PG cell proliferation *in vitro* when added directly to the cultured medium (Table 2); but *Gl*-BSP (50, 100, and 200 mg kg^−1^)-treated serum of S180-bearing mice markedly inhibited S180 or PG cell proliferation (Figure 1).

**Table 2:** **Effect of *Gl*-BSP on proliferation of Sarcoma 180 cells and PG cells *in vitro* ($\bar{x}\pm \text{s}$)**.

Group	Concentration (mg l^−1^)	Inhibitory ratio (%)	
		Sarcoma 180 cells	PG cells
		(*n* = 7)	(*n* = 5)
RMPI-1640	–	0.0	0.0
*Gl*-BSP	0.1	0.2	1.4
	1	3.1	0.2
	10	1.3	0.4
	100	3.3	2.0
	400	7.1	0.8

### Effect of *Gl*-BSP on spleen lymphocyte proliferation induced by con A and LPS in S180-bearing mice

Compared with normal mice, proliferation of spleen lymphocyte induced by Con A and LPS declined significantly in S180-bearing mice administrated with physiological saline (model group), but *Gl*-BSP administrated intragastrically promoted spleen lymphocytes proliferation induced by Con A and LPS in S180-bearing mice (Table 3).

**Table 3:** **Effect of *Gl*-BSP on spleen lymphocyte proliferation induced by Con A and LPS in S180-bearing mice (*n* = 5, $\bar{x}\pm \text{s}$)**.

Group	Dose (mg kg^−1^ × days)	Proliferation ratio (%)	
		Con A (1 mg l^−1^)	LPS (5 mg l^−1^)
Normal	–	254.0 ± 8.4	127.0 ± 8.8
Model	–	130.3 ± 8.3^###^	108.8 ± 7.6^##^
CY	30 mg kg^−1^ × 7 (i.p, q.o.d)	105.0 ± 2.1***	71.2 ± 5.7***
*Gl*-BSP	50 mg kg^−1^ × 14	184.3 ± 3.4***	115.5 ± 8.9
	100 mg kg^−1^ × 14	230.5 ± 10.2***	116.8 ± 0.5
	200 mg kg^−1^ × 14	236.4 ± 6.7***	123.4 ± 5.7*

S180-bearing mice were administrated intragastrically with *Gl*-BPS (50, 100, or 200 mg kg^−1^) or physiological saline respectively for 14 days. At the end of experiment, single splenic lymphocytes were adjusted to a concentration of 1 × 10^6^ cells per well in 96-well culture plates with or without ConA 1 mg l^−1^ or LPS 5 mg l^−1^, and incubated at 37°C, 5% CO_2_ for 72 h. Cell proliferation was estimated based on the method of MTT. ^##^*P* < 0.01, ^###^*P* < 0.001 model group vs. normal group; *P < 0.05, ***P < 0.001 vs. model group.

### Effect of *Gl*-BSP on splenic NK cytotoxic activity in S180-bearing mice

Compared with normal mice, splenic NK cytotoxic activity in model group was significantly lower, reduced from 35.6% down to 8.3%, while *Gl*-BSP (50, 100, and 200 mg kg^−1^) administrated intragastrically increased the splenic NK cytotoxic activity of S180-bearing mice (Table 4).

**Table 4:** **Effect of *Gl*-BSP on splenic NK cytotoxic activity in S180-bearing mice (*n* = 5, $\bar{x}\pm \text{s}$)**.

Group	Dose (mg kg^−1^ × days)	Cytotoxicity (%)
Normal	–	35.6 ± 2.3
Model	–	8.3 ± 1.7^###^
CY	30 mg kg^−1^ × 7 (i.p, q. o. d)	0.6 ± 0.5***
*Gl*-BSP	50 mg kg^−1^ × 14	13.8 ± 1.0**
	100 mg kg^−1^ × 14	21.4 ± 2.9***
	200 mg kg^−1^ × 14	30.3 ± 1.1***

S180-bearing mice were administrated intragastrically with *Gl*-BPS (50, 100, or 200 mg kg^−1^) or physiological saline for 14 days, and the splenic NK cytotoxic activity of mice was examined in a 4-h ^51^Cr assay. Target cell: YAC-1. Effector: target cell = 40:1; ^###^*P* < 0.001 model group vs. normal group; **P < 0.01, ***P < 0.001 vs. model group.

### Effect of *Gl*-BSP on phagocytosis by peritoneal macrophages in S180-bearing mice

*Gl*-BSP (50, 100, or 200 mg kg^−1^) could augment the percentage of phagocytosis of NR by peritoneal macrophages in S180-bearing mice (Table 5).

**Table 5:** **Effect of *Gl*-BSP on phagocytosis by peritoneal macrophages in S180-bearing mice (*n* = 5, $\bar{x}\pm \text{s}$)**.

Group	Dose (mg kg^−1^ × days)	Phagocytosis ratio (%)
Normal	–	100.1 ± 11.0
Model	–	48.2 ± 5.0^###^
*Gl*-BSP	50 mg kg^−1^ × 14	75.0 ± 4.0**
	100 mg kg^−1^ × 14	134.1 ± 7.8***
	200 mg kg^−1^ × 14	141.6 ± 27.8***

S180-bearing mice were administrated intragastrically with *Gl*-BPS (50, 100, or 200 mg kg^−1^) or physiological saline respectively for 14 days. At the end of experiment, the activity of peritoneal macrophages was measured by NR phagocytosis assay. ^###^*P* < 0.001 model group vs. normal group; **P < 0.01, ***P < 0.001 vs. model group.

### Effect of *Gl-*BSP on T lymphocyte subpopulation in S180-bearing mice

Compared with normal mice, the percentage of the CD4^+^ or CD8^+^ subset decreased markedly, while the CD4^+^/CD8^+^ ratio increased significantly in the model group. The results indicated that the number of CD8^+^ subset decreased more than that of CD4^+^ subset. Compared with the model group, the percentage of the CD4^+^ and CD8^+^ subset in S180-bearing mice treated with *Gl*-BSP (50, 100, or 200 mg kg^−1^) was greatly augmented, and the ratio of CD4^+^/CD8^+^ reduced nearly to that in normal mice (Table 6).

**Table 6:** **Effect of *Gl-*BSP on T lymphocyte subpopulation in S180-bearing mice (*n* = 3, $\bar{x}\pm \text{s}$)**.

Group	Dose (mg kg^−1^ × days)	CD4^+^ (%)	CD8^+^ (%)	CD4^+^/CD8^+^
Normal	–	33.67 ± 0.94	18.14 ± 1.46	1.86 ± 0.11
Model	–	20.98 ± 0.26^###^	9.46 ± 0.48^###^	2.22 ± 0.12^###^
*Gl*-BSP	50 mg kg^−1^ × 14	22.73 ± 0.31**	11.27 ± 0.55**	2.02 ± 0.09*
	100 mg kg^−1^ × 14	22.99 ± 0.92**	11.95 ± 0.64***	1.93 ± 0.03**
	200 mg kg^−1^ × 14	23.81 ± 1.08***	12.14 ± 0.05***	1.96 ± 0.10**

S180-bearing mice were administrated intragastrically with *Gl*-BPS (50, 100, or 200 mg kg^−1^) or physiological saline respectively for 14 days; single splenic lymphocytes were labeled with anti-mouse CD4-FITC and anti-mouse CD8-PE. The CD4 and CD8 T-cell subpopulations were analyzed by flow cytometry using a FACS Calibur. ^###^*P* < 0.001 model group vs. normal group; *P < 0.05, **P < 0.01, ***P < 0.001 vs. model group.

### Effect of *Gl*-BSP on production of serum cytokines in S180-bearing mice

On the basis of the effect of *Gl*-BSP on splenic lymphocytes, NK cells, and peritoneal macrophages, the production of serum cytokines was detected. We found that IL-2, IFN-γ, and TNF-α in serum were undetectable in normal mice. Only IFN-γ in serum was detectable in S180-bearing mice. The serum levels of IL-2, IFN-γ, and TNF-α were markedly increased in S180-bearing mice administrated with *Gl*-BSP (200 mg kg^−1^), compared with that in S180-bearing control mice administrated with physiological saline (Table 7).

**Table 7:** **Effect of *Gl*-BSP on Production of Serum Cytokines and NO in S180-bearing mice (*n* = 10, $\bar{x}\pm \text{s}$)**.

Group		IL-2 (pg ml^−1^)	IFN-γ (pg ml^−1^)	TNF-α (pg ml^−1^)	NO^2−^ (μM)
Normal control	–	UD	UD	UD	6.78 ± 4.94
S180-bearing control	–	UD	11.73 ± 3.53	UD	4.82 ± 3.74
*Gl*-BSP	50 mg kg^−1^	UD	13.24 ± 3.48	UD	12.97 ± 2.45
	100 mg kg^−1^	UD	20.13 ± 2.93**	20.62 ± 16.8	17.32 ± 5.30**
	200 mg kg^−1^	3.4 ± 2.46***	67.42 ± 5.47***	72.58 ± 33.40***	35.63 ± 10.0***

S180-bearing mice were administrated intragastrically with *Gl*-BPS (50, 100, 200 mg kg^−1^) or physiological saline respectively for 14 days. At the end of experiment, the serum was collected and the production of serum cytokines was detected by ELISA. Serum NO level was measured by Griess reagent. **P < 0.01, ***P < 0.001 vs. S180-bearing control; UD: undetected.

### Antibody neutralization

To determine whether the growth inhibition in Sarcoma 180 cells or PG cells induced by *Gl*-BSP 200 mg kg^−1^-treated serum was related to the cytokines released from immune system activated by *Gl*-BSP in S180-bearing tumor mice. Serums collected from mice in all groups were preincubated with one or two cytokine-neutralizing antibodies before addition to cell cultures. Neutralization with anti-TNF-α apparently diminished S180 or PG cell lines growth inhibition induced by *Gl*-BSP 200 mg kg^−1^-treated serum, and a similar result was obtained by use of anti-IFN-γ. Blocking effect was noted in the combination of anti-TNF-α and anti-IFN-γ, which reduced the inhibition rate from 55.6 or 45.1% (before neutralization in S180 or PG cell lines) down to 11 or 15.8%, respectively. However, the cytokine antibodies used above did not completely block growth inhibition induced by the *Gl*-BSP 200 mg kg^−1^-treated serum of S180-bearing mice (Table 8).

**Table 8:** **Effect of *Gl*-BSP-treated serum of S180-bearing mice preincubated with cytokine-neutralizing antibodies on the proliferation of S180 and PG cells (*n* = 10, $\bar{x}\pm \text{s}$)**.

Group	Inhibitory ratio (%)	
	S180	PG
*Gl*-BSP 200 mg kg^−1^-treated serum alone	55.6 ± 1.7	45.1 ± 2.4
*Gl*-BSP 200 mg kg^−1^-treated serum + anti-TNF-α	32.5 ± 1.2***	29.0 ± 4.0***
*Gl*-BSP 200 mg kg^−1^-treated serum + anti-IFN-γ	45.0 ± 2.4***	34.1 ± 3.0***
*Gl*-BSP 200 mg kg^−1^-treated serum + anti-TNF-α + anti-IFN-γ	11.3 ± 0.9***	15.8 ± 3.3***

*Gl*-BSP 200 mg kg^−1^-treated serum of S180-bearing mice preincubated with or without various cytokine-neutralizing antibodies (anti-TNF-α, 150 ng ml^−1^, and/or anti-IFN-γ, 150 ng ml^−1^) at 37°C for 90 min before addition to S180 or PG cells cultures. Data are the mean ± SD of 3 separate experiments. ***P < 0.001 vs. *Gl*-BSP 200 mg kg^−1^-treated serum.

### Effect of *Gl*-BSP-treated serum with or without heat inactivation on proliferation of S180 cells *in vitro*

**Figure 2:**
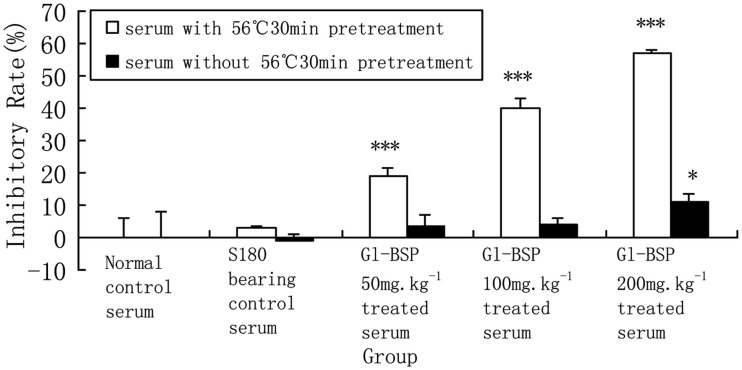
**Effect of *Gl*-BSP-treated serum with or without 30 min 56°C heat pretreatment on proliferation of S180 cells *in vitro* (*n* = 10, $\bar{x}\pm \text{s}$)**. *P < 0.05, **P < 0.01, ***P < 0.001 vs. normal control.

We found that the *Gl*-BSP-treated serum of S180-bearing mice had the different effects on Sarcoma 180 cell proliferation *in vitro*. After *Gl*-BSP 200 mg kg^−1^-treated serum underwent heat-inactive at 56°C for 30 min, the inhibition rate reduced from 57.1% down to 10.9%; while *Gl*-BSP 50 mg kg^−1^ and 100 mg kg^−1^-treated serum with pretreatment at 56°C for 30 min did not show significantly effect on the proliferation of Sarcoma 180 cells *in vitro* (Figure 2).

## Discussion

In the present study, we investigated the antitumor activity of the polysaccharides isolated from broken-spore of *G. lucidum*, *Gl*-BSP. The spore of *G. lucidum* has a especially tough wall. Recently some researchers indicated that broken spores have more activities than non-broken spores (Zhu et al., 2000; Sliva et al., 2003; Wang et al., 2005). In our research, we also compared the antitumor and immunomodulatory effects of non-broken spore polysaccharides (*Gl*-SP) and *Gl*-BSP extracted from *G. lucidum*. We found that both *Gl*-SP and *Gl*-BSP could inhibit the tumor growth and improve the immune function in tumor-bearing mice *in vivo*, but *Gl*-BSP showed much higher bioactivities than *Gl*-SP (data not showed).

β-Glucans are one of the most abundant forms of polysaccharides found inside the cell wall of bacteria and fungus. All β-glucans are glucose polymers linked together by a 1 → 3 linear β-glycosidic chain core and they differ from each other by their length and branching structures. Fungal β-glucan is β-d-glucose linked to one another by 1 → 3 glycosidic chain with 1 → 6 glycosidic branches whereas bacterial β-glucans have 1 → 4 side branches. Bacterial endotoxin such as lipopolysaccharide (LPS) is a potent immune stimulator and exogenous pyrogen, which causes fever, shock, and disseminated intravascular coagulation. Endotoxin contamination can lead to false positive results in immune tests and toxicity (Chan et al., 2009; Gertsch et al., 2011). Hence, we evaluated the quantity of endotoxin in *Gl*-BSP by limulus amebocytes lysate assay, which was less than 0.01 EU mg^−1^. This data indicated that endotoxin contamination in *Gl*-BSP used in this study was negligible. It should be noted that *Gl*-BSP is a complex consisted of polysaccharides and peptide, but endotoxin has no peptide. It is therefore possible that peptide in *Gl*-BSP may be involved in some of the bioactivity of *Gl*-BSP. Furthermore, there was no evident of toxic or side effect observed in *Gl*-BSP treated mice during our experiments.

Kuang et al. (2007) used *G. lucidum* broken-spore powder combined with chemotherapy in the treatment of 28 patients of malignant neoplastic diseases. The patients were orally treated with 0.9 g of *G. lucidum* broken-spore at a time, three times a day, for 6 weeks, combined with chemotherapy at the same time. Qi et al. (1999) used *G. lucidum* spore powder combined with chemotherapy in the treatment of 100 patients of malignant tumor. The patients were treated with 1 g of *G. lucidum* spore at a time, three times a day, for a month, combined with chemotherapy at the same time. The above clinical trials demonstrated that *G. lucidum* spore’s adjuvant efficacy in treating cancer patients by strengthening the immunity and decreasing toxic side effects caused by chemotherapy, compared with chemotherapy alone.

Extraction ratio of polysaccharides from *G. lucidum* spore is about 2.4% (Lin et al., 2003). A dose of *G. lucidum* polysaccharides about 0.07 g per day in human is equal to about 10 mg kg^−1^ per day in mice, according to the equivalent conversion of dose between species (Mizuno et al., 1995). *G. lucidum* is approved by Ministry of Health in China as a new drug resource. Thus, less than 100 mg kg^−1^ *Gl*-BSP may represent a real dose and further study is needed to confirm this assumption.

The present results showed that *Gl*-BSP inhibited the growth of implanted Sarcoma 180 markedly. On the basis of the preceding *in vivo* results, we examined the direct *in vitro* effects of *Gl*-BSP or serum from *Gl*-BSP treated mice in Sarcoma 180 and PG cell cultures. We found that *Gl*-BSP could not inhibit their proliferation *in vitro* indicating that *Gl*-BSP had no direct cytotoxicity on tumor cells. In contrast, *Gl*-BSP (50, 100, or 200 mg kg^−1^)-treated serum could significantly inhibit Sarcoma 180 cells and PG cells proliferation. Our previous studies imply that *Gl*-BSP can potentiate immunomodulatory activity *in vivo* that contributes significantly to its antitumor activity (Wang et al., 2005).

We investigated the effect of *Gl*-BSP on splenic lymphocyte, spleen NK cell and peritoneal macrophage activities in S180-bearing BALB/c mice *in vivo*. The results *in vivo* were consistent with those *in vitro*. *Gl*-BSP promoted the spleen lymphocytes proliferation induced by Con A or LPS, enhanced NK cytotoxic activity, augmented the percentage of phagocytosis of NR by macrophages, and increased the percentage of the CD4^+^ or CD8^+^ subset in S180-bearing BALB/c mice. *Gl*-BSP showed immunomodulatory activities in improving the immuno-repressive state of tumor-bearing mice. The mean serum concentration of TNF-α, IFN-γ and NO apparently increased in *Gl*-BSP-treated groups. TNF-α and IFN-γ play important roles in depressing tumor cell growth and inducing apoptosis of tumor cells (Spanaus et al., 1998; Smyth and Johnstone, 2000; Shin et al., 2001; Ruiz de Almodovar et al., 2004). They are potential endogenous products in mediating the effect of *Gl*-BSP on the immune system *in vivo*. Anti-TNF-α apparently diminished the growth inhibitory effect of serum from *Gl*-BSP 200 mg kg^−1^-treated S180-bearing mice in S180 or PG cell lines. Similar results were obtained using anti-IFN-γ. Greater neutralization effect was noted in the combination of anti-TNF-α and anti-IFN-γ in reducing the inhibition rate of serum from *Gl*-BSP 200 mg kg^−1^-treated S180-bearing mice (55.6% before neutralization to 11.3% in S180 cell lines and from 45.1% before neutralization to 15.8% in PG cell lines respectively). However, the cytokine antibodies used above did not completely block growth inhibition of serum from *Gl*-BSP 200 mg kg^−1^-treated S180-bearing mice in S180 or PG cell lines suggesting other bioactive products in the serum, such as NO and complements may also play a role.

There may be other active ingredients in the serum from *Gl*-BSP-treated mice, such as NO, activated complement C3 and metabolized substance of *Gl*-BSP. NO has been identified as the major effector molecule involved in the destruction of tumor cells by activated macrophages (Stuehr and Nathan, 1989; Keller et al., 1990). Also, NO has been recognized as an important messenger in diverse pathophysiological functions, including neuronal transmission, vascular relaxation, immune modulation, and cytotoxicity against tumor cells (Lowenstein et al., 1994). NO being a gaseous free radical, has a short half life and is rapidly metabolized to nitrate (${\text{NO}}_{\text{3}}^{\text{−}}$) and nitrite (${\text{NO}}_{\text{2}}^{\text{−}}$). So it is difficult to measure NO directly, nitrate or nitrite are measured normally as indices of NO production (Coleman, 2001). We observed the mean serum concentration of NO increased in *Gl*-BSP -treated groups, especially in *Gl*-BSP 200 mg kg^−1^-treated group. As a result, NO in serum might be another inhibitory component in suppressing the growth of Sarcoma 180 or PG cells *in vitro*. Considering the activity of cytokines and NO, serum derived from all tested mice did not be heat-inactive at 56°C for 30 min in the former experiments. We compared the different effect on proliferation of S180 cells *in vitro* between *Gl*-BSP-treated serum with and without pretreatment at 56°C for 30 min. The result showed some activated substances lost theirs activity after *Gl*-BSP-treated serum underwent heat-inactive at 56°C for 30 min.

Although effect of humoral immunity to tumor is not as important as that of cellular immune responses against tumor, IgM and IgG1 (IgG3) which might be in *Gl*-BSP-treated serum could mediate cytolytic activity with the help of component C3. A polysaccharide isolated from spores of *G. lucidum* showed significant effect in enhancing the serum component C3 level in mice but had a small effect in increasing the production of antibodies against sheep red blood cells in mice (Bao et al., 2001a). Further investigation is necessary to determine whether or not the effect of *Gl*-BSP on the complement system and humoral immune responses may also contribute to the antitumor mechanism of *Gl*-BSP.

In summary, we demonstrated that *Gl*-BSP inhibited the tumor growth and restored the depressed immune response in tumor-burdened mice to the normal or near normal level. The antitumor activity of *Gl*-BSP may be related to the activation of the immune response of the host organism by the stimulation of NK cells, T cells, and macrophage-dependent immune system responses. Our results provide a clue that *Gl*-BSP may be considered as an efficacious adjacent immunopotentiating therapy in the treatment of tumor where immunosuppression occurs. Further studies are required to elucidate and confirm the effectiveness of restoring the immune response by *GL-BSP* in the treatment of tumor.

## Conflict of Interest Statement

The authors declare that the research was conducted in the absence of any commercial or financial relationships that could be construed as a potential conflict of interest.

## References

[B1] Bao X. Duan J. Fang X. Fang J. (2001a). Chemical modifications of the (1 → 3)-alpha-d-glucan from spores of *Ganoderma lucidum* and investigation of their physicochemical properties and immunological activity. Carbohydr. Res. 336, 127–140 10.1016/S0008-6215(01)00238-5 11689183

[B2] Bao X. Fang J. Li X. (2001b). Structural characterization and immunomodulating activity of a complex glucan from spores of *Ganoderma lucidum*. Biosci. Biotechnol. Biochem. 65, 2384–2391 10.1271/bbb.65.2384 11791709

[B3] Bao X. F. Fang J. N. (2001). Studies on difference between sporoderm-broken and nonbroken spores of *Ganoderma lucidum* (Leyss. ex Fr.) Karst. by polysaccharide analysis. Zhong guo Zhong Yao Za Zhi 26, 326–328 12528523

[B4] Bao X. F. Dong Q. Fang J. N. (2000). Structure and conformation behavior of a glucan from spores of *Ganoderma lucidum* (Fr.) Karst. Sheng Wu Hua Xue Yu Sheng Wu Wu Li Xue Bao 32, 557–561 12058164

[B5] Chan G. C. F. Chan W. K. Sze D. M. Y. (2009). The effects of β-glucan on human immune and cancer cells. J. Hematol. Oncol. 2, 25 10.1186/1756-8722-2-25 19515245 PMC2704234

[B6] Coleman J. W. (2001). Nitric oxide in immunity and inflammation. Int. Immunopharmacol. 1, 1397–1406 10.1016/S1567-5769(01)00085-6 11515807

[B7] Gertsch J. Viveros-Paredes J. M. Taylor P. (2011). Plant immunostimulants-scientific paradigm or myth? J. Ethnopharmacol. 136, 385–391 10.1016/j.jep.2010.06.044 20620205

[B8] Hsu M. J. Lee S. S. Lee S. T. Lin W. W. (2003). Signaling mechanisms of enhanced neutrophil phagocytosis and chemotaxis by the polysaccharide purified from *Ganoderma lucidum*. Br. J. Pharmacol. 139, 289–298 10.1038/sj.bjp.0705243 12770934 PMC1573843

[B9] Hsu M. J. Lee S. S. Lin W. W. (2002). Polysaccharide purified from *Ganoderma lucidum* inhibits spontaneous and Fas-mediated apoptosis in human neutrophils through activation of the phosphatidylinositol 3 kinase/Akt pathways. J. Leuokoc. Biol. 72, 207–216 12101282

[B10] Hu Y. H. Lin Z. B. (1999). Effects of polysaccharides isolated from mycelia of *Ganoderma lucidum* on HL-60 cell apoptosis. Acta Pharmacol. Sin. 34, 264–268

[B11] Keller R. Keist R. Wechsler A. Leist T. P. van der Meide P. H. (1990). Mechanisms of macrophage-mediated tumor cell killing: a comparative analysis of the roles of reactive nitrogen intermediates and tumor necrosis factor. Int. J. Cancer 46, 682–686 10.1002/ijc.2910460422 2120138

[B12] Kim H. S. Kacew S. Lee B. M. (1999). In vitro chemopreventive effects of plant polysaccharides (Aloe barbadendis miller, Lentis edodes, *Ganoderma lucidum* and *Coriolus versicolor*). Carcinogenesis 20, 1637–1640 10.1093/carcin/20.8.1637 10426820

[B13] Kuang J. M. Wang J. W. Kuang J. M. (2007). Curative effects of combination of *Ganoderma lucidum* broken-spore powder and chemotherapy on malignant tumors. Shandong Med. J. 47, 59–60

[B14] Lee S. H. Lee P. L. Chen C. F. Wang S. Y. Chen K. Y. (2003). Antitumor effects of polysaccharides of *Ganoderma lucidum* (Curt.: Fr.) P. Karst. (Ling Zhi, Reishi Mushroom) (Aphyllphoromycetideae). Int. J. Med. Mushrooms 2003, 1–16 10.1615/InterJMedicMush.v5.i1.10

[B15] Lin S. Q. Wang S. Z. Lin Z. B. Lin Y. X. (2003). Isolation and identification of active components of *Ganoderma lucidum* cultivated with grass and wood log I. Extraction, purification and characterization of glycopeptides. Chin. Tradit. Herb. Drugs 34, 33–35

[B16] Lin Z. B. (2001). Modern Research of Ganoderma lucidum, 2nd Edn. Beijing: Beijing Medical University Press, 219–283

[B17] Lin Z. B. Zhang H. N. (2004). Antitumor and immunoregulatory activities of *Ganoderma lucidum* and its possible mechanisms. Acta Pharmacol. Sin. 25, 1387–1395 15525457

[B18] Liu G. T. (1999). Pharmacology and clinical application of the spores of *Ganoderma lucidum* (Curt:Fr.) P. Karst. and mycelium of *Ganoderma capense* (Lloyd) Teng. (Aphyllophromcitideae). Int. J. Med. Mushrooms 1, 63–67

[B19] Liu X. Yuan J. P. Chung C. K. Chen X. J. (2002). Antitumor activity of the sporoderm-broken germinating spores of *Ganoderma lucidum*. Cancer Lett. 182, 155–161 10.1016/S0304-3835(02)00080-0 12048161

[B20] Lowenstein C. F. Dinerman J. L. Snyder S. H. (1994). Nitric oxide: a physiologic messenger. Ann. Intern. Med. 120, 227–237 8273987 10.7326/0003-4819-120-3-199402010-00009

[B21] Lu Q. Y. Jin Y. S. Zhang Q. Zhang Z. Heber D. Go V. L. Li F. P. Rao J. Y. (2004). *Ganoderma lucidum* extracts inhibit growth and induce actin polymerization in bladder cancer cells in vitro. Cancer Lett. 16, 9–20 15500944 10.1016/j.canlet.2004.06.022

[B22] Miyazaki T. Nishijima M. (1981). Studies on fungal polysaccharides. XXVII. Structural examination of a water-soluble, antitumor polysaccharide of *Ganoderma lucidum*. Chem. Pharm. Bull. 29, 3611–3616 10.1248/cpb.29.883 7340947

[B23] Mizuno T. Sakai T. Chihara G. (1995). Health foods and medicinal usages of mushrooms. Food Rev. Int. 11, 69–81 10.1080/87559129509541017

[B24] Qi Y. F. Li X. R. Yan M. Liu A. Jiao Z. H. Liu Y. (1999). Clinical observation of treating digestive system tumor with *Ganoderma lucidum* spore powder as adjuvant chemotherapy. Chin. J. Integr. Med. 19, 554–555

[B25] Ruiz de Almodovar C. Lopez-Rivas A. Ruiz-Ruiz C. (2004). Interferon-gamma and TRAIL in human breast tumor cells. Vitam. Horm. 67, 291–318 10.1016/S0083-6729(04)67016-6 15110183

[B26] Sakagami H. Aoki T. Simpson A. Tanuma S. (1991). Induction of immunopotentiation activity by a protein-bound polysaccharide, PSK. Anticancer Res. 11, 993–999 2064356

[B27] Shin E. C. Ahn J. M. Kim C. H. Choi Y. Ahn Y. S. Kim H. Kim S. J. Park J. H. (2001). IFN-gamma induces cell death in human hepatoma cells through a TRAIL/death receptor-mediated apoptotic pathway. Int. J. Cancer 93, 262–268 10.1002/ijc.1310 11410875

[B28] Sliva D. Labarrere C. Slivova S. Sedlak M. Lloyd F. P. Jr. Ho N. W. (2002). *Ganoderma lucidum* suppresses motility of highly invasive breast and prostate cancer cells. Biochem. Biophys. Res. Commun. 298, 603–612 12408995 10.1016/s0006-291x(02)02496-8

[B29] Sliva D. Sedlak M. Slivova V. Valachovicova T. Lloyd F. P. Jr. Ho N. W. (2003). Biologic activity of spores and dried powder from *Ganoderma lucidum* for the inhibition of highly invasive human breast and prostate cancer cells. J. Altern. Complement. Med. 9, 491–497 10.1089/107555303322284776 14499024

[B30] Smyth M. J. Johnstone R. W. (2000). Role of TNF in lymphocyte-mediated cytotoxicity. Microsc. Res. Tech. 50, 196–208 10.1002/1097-0029(20000801)50:3<196::AID-JEMT3>3.0.CO;2-9 10891885

[B31] Sone Y. Okuda R. Wada N. Kishida E. Misaki A. (1985). Structure and antitumor activities of polysaccharides isolated from fruiting body and the growing culture of mycelium of *Ganoderma lucidum*. Agric. Biol. Chem. 49, 2641–2653 10.1271/bbb1961.49.2641

[B32] Spanaus K. S. Schlapbach R. Fontana A. (1998). TNF-α and IFN-γ render microglia sensitive to Fas ligand-induced apoptosis by induction of Fas expression and down-regulation of Bcl-2 and Bcl-xL. Eur. J. Immunol. 28, 4398–4408 10.1002/(SICI)1521-4141(199812)28:12<4398::AID-IMMU4398>3.0.CO;2-Y 9862377

[B33] Stuehr D. J. Nathan C. F. (1989). Nitric oxide: a macrophage product responsible for cytostasis and respiratory inhibition in tumor target cells. J. Exp. Med. 169, 1543–1555 10.1084/jem.169.5.1543 2497225 PMC2189318

[B34] Susan W. S. Leung K. Y. Yeung Y. L. Siow R. Man Y. K. (2002). “Lingzhi (Ganoderma) research – the past, present and future perspectives,” in Ganoderma: Genetics, Chemistry, Pharmacology, and Therapeutics, ed. Lin Z. B. (Beijing: Beijing Medical University Press), 1–9

[B35] Wang P. Y. Wang S. Z. Lin S. Q. Lin Z. B. (2005). Comparison of the immunomodulatory effects of spore polysaccharides and broken spore polysaccharides isolated from *Ganoderma lucidum* on murine splenic lymphocytes and peritoneal macrophages in vitro. Beijing Da Xue Xue Bao 37, 569–574 16378103

[B36] Wang S. Y. Hsu M. L. Hsu H. C. Tzeng C. H. Lee S. S. Shiao M. S. Ho C. K. (1997). The anti-tumor effect of *Ganoderma Lucidum* is mediated by cytokines released from activated macrophages and T lymphocytes. Int. J. Cancer 70, 699–705 10.1002/(SICI)1097-0215(19970317)70:6<699::AID-IJC12>3.0.CO;2-5 9096652

[B37] Wang Y. Y. Khoo K. H. Chen S. T. Lin C. C. Wong C. H. Lin C. H. (2002). Studies on the immuno-modulating and antitumor activities of *Ganoderma lucidum* (Reishi) polysaccharides: functional and proteomic analyses of a fucose-containing glycoprotein fraction responsible for the activities. Bioorg. Med. Chem. 10, 1057–1062 10.1016/S0968-0896(01)00315-7 11836115

[B38] Wasser S. P. (2002). Medicinal mushrooms as a source of antitumor and immunomodulating polysaccharides. Appl. Microbiol. Biotechnol. 60, 258–274 10.1007/s00253-002-1076-7 12436306

[B39] Xia D. Lin Z. B. Li R. Z. He Y. Q. (1989). Effects of *Ganoderma* polysaccharides on immune function in mice. Yao Xue Xue Bao. 21, 533–537

[B40] Yan J. Vetvicka V. Xia Y. Coxon A. Carroll M. C. Mayadas T. N. Ross G. D. (1999). Beta-glucan, a “specific” biologic response modifier that uses antibodies to target tumors for cytotoxic recognition by leukocyte complement receptor type 3 (CD11b/CD18). J. Immunol. 163, 3045–3052 10477568

[B41] Yang X. L. Zhu H. S. Xu J. L. Kuang Q. (1997). Antitumor activity of the *Ganoderma Lucidum* spore alcohol extract in vitro. J. Beijing Inst. Technol. 6, 336–340

[B42] You Y. H. Lin Z. B. (2002). Protective effects of Ganoderma lucidum polysaccharides peptide on injury of macrophages induced by reactive oxygen species. Acta Pharmacol. Sin. 23, 787–791 12230945

[B43] Zhang G. L. Wang Y. H. Ni W. Teng H. L. Lin Z. B. (2002). Hepatoprotective role of Ganoderma lucidum polysaccharide against BCG-induced immune liver injury in mice. World J. Gastroenterol. 8, 728–733 12174387 10.3748/wjg.v8.i4.728 PMC4656329

[B44] Zhang H. N. Lin Z. B. (2003). Prevention of low-dose of strptozotocin-induced autoimmune diabetic mice with Ganoderma lucidum polysaccharides. Natl. Med. J. China. 83, 1999–2000

[B45] Zhang Q. H. Lin Z. B. (1999). The antitumor activity of *Ganoderma lucidum* polysaccharides is related to tumor necrosis factor-α and interferon-γ. Int. J. Med. Mushrooms 1, 207–215

[B46] Zhu H. S. Yang X. L. Wang L. B. Zhao D. X. Chen L. (2000). Effects of extracts from sporoderm-broken spores of *Ganoderma lucidum* on HeLa cells. Cell Biol. Toxicol. 16, 201–206 10.1023/A:1007663006548 11032363

